# Feasibility of an inertial measurement unit sensor-based guiding system for benign paroxysmal positional vertigo treatment: A pilot study

**DOI:** 10.1038/s41598-023-29685-8

**Published:** 2023-02-23

**Authors:** Cecilia A. Callejas Pastor, Chiheon Kwon, Jung Sook Joo, Hee Chan Kim, Dae Bo Shim, Yunseo Ku, Myung-Whan Suh

**Affiliations:** 1 Department of Biomedical Engineering, College of Medicine, Chungnam National University, Munhwa-ro 266, Jung-gu, Daejeon, 35016 Republic of Korea; 2 Department of Biomedical Research Institute, Chungnam National University Hospital, Daejeon, Republic of Korea; 3 Department of Otorhinolaryngology-Head and Neck Surgery, Seoul National University Hospital, Yongon-dong, Chongno-gu, Seoul, 110-744 Republic of Korea; 4 Interdisciplinary Program in Bioengineering, Graduate School, Seoul National University, Seoul, Republic of Korea; 5 Institute of Medical and Biological Engineering, Medical Research Center, Seoul National University, Seoul, Republic of Korea; 6 Department of Biomedical Engineering, College of Medicine, Seoul National University, Seoul, Republic of Korea; 7 Department of Otorhinolaryngology, Myongji Hospital, Hanyang University College of Medicine, Goyang, Republic of Korea

**Keywords:** Biomedical engineering, Electrical and electronic engineering, Software

## Abstract

Performing an accurate canalith repositioning procedure (CRP) is important for treating benign paroxysmal positional vertigo, because inadequate rotational head angles can result in ineffective otolith mobilization and consequent treatment failure. Specialists-guided Epley maneuver reportedly had mean errors of 13.7°–24.4° while they were significantly larger (40.0°–51.5°) when self-administered. Similar results were obtained for the Barbeque maneuver: mean errors were 9.2°–13.0° by the specialists while they were significantly larger (22.9°–28.6°) when self-administered. Our study aimed to validate the feasibility of an inertial measurement unit sensor-based CRP (IMU-CRP) by analyzing the differences in accuracy in the rotational angles, comparing them with education-based conventional CRP (EDU-CRP). A pilot validation was also performed by analyzing the treatment success rate of IMU-CRP in patients with BPPV. This single-institution prospective, comparative effectiveness study examined 19 participants without active vertigo or prior knowledge of benign paroxysmal positional vertigo and CRP. Participants conducted the Epley and Barbeque roll maneuvers without and with auditory guidance (EDU-CRP vs. IMU-CRP, respectively) twice, and head rotation accuracies were compared. Differences in target angles based on the American Academy of Otolaryngology-Head and Neck Surgery guidelines were considered errors. For BPPV participants, treatment success was assessed based on the presence or absence of nystagmus, vertigo, and dizziness. For all the Epley and Barbeque roll maneuvers steps, the absolute errors were smaller for IMU- than for EDU-CRPs, with significant differences in steps 2–4 and 3–6 of the Epley and Barbeque roll maneuvers, respectively. A learning effect was found in steps 4 and 5 of the Barbeque roll maneuver but not in the Epley maneuver. The treatment success rates after 1 h were 71.4% and 100% for the Epley and Barbeque roll maneuvers, respectively. Real-time feedback on head rotation angles induced more appropriate movements in the Epley and Barbeque roll maneuvers. A guiding device based on head monitoring providing real-time auditory feedback may increase the self-administered CRP success rates in treating benign paroxysmal positional vertigo.

## Introduction

Benign paroxysmal positional vertigo (BPPV) can be treated by a series of rotational head movements geometrically aligned with the affected semicircular canal. A symptom reduction in patients with BPPV with posterior canal involvement is 3.3–107.7 times more likely following a canalith repositioning procedure (CRP) than under control conditions^1–4^. However, only 58% of patients with BPPV were discharged following the first CRP, whereas 94% needed up to three follow-up appointments^5^. Repeated CRPs at home can improve treatment outcomes. A home CRP program including Brandt–Daroff, Semont, 360° rotation, and deep head hanging maneuvers following conventional office-based CRP was 8.3% more effective than office-based CRP alone^6^. Home CRP also reduced BPPV recurrence by 9%^7^. Therefore, repeated or self-administered CRP can support effective BPPV care.

Self-administered CRPs showed significantly higher errors in rotational head angles compared to specialists-guided CRPs adhering to American Academy of Otolaryngology-Head and Neck Surgery (AAO-HNS) guidelines^8,9^. Without specialist assistance, patients have difficulties performing precise CRP maneuvers due to the complexity of sequential 3-dimensional (3-D) head movements. In the Epley maneuver, the absolute mean error was as large as 39.8° in step 4, even after detailed explanations. The comparatively simpler Barbeque (BBQ) roll maneuver still caused substantial rotation errors, especially in elderly participants, the population mostly affected by BPPV^8^. Patients can also perform CRP using only audiovisual media such as YouTube. However, their reliability can be as low as 64%^10^, and watching a well-made video clip does not guarantee well-performed CRPs. One of the reasons is that video clips cannot provide feedback on the accuracy of head movements or how to correct them in real-time.

The present study aimed to validate the feasibility of an inertial measurement unit (IMU) sensor-based CRP guiding system that provides step-by-step instructions and real-time feedback while participants perform CRP independently. Epley and BBQ roll maneuvers were performed, and the 3-D rotational head movements were quantitatively tracked with head-mounted IMU sensors. The rotational angle errors were compared between education-based conventional (EDU)-CRP and IMU-CRP. Learning effects were also investigated with test–retest sessions. Finally, we analyzed the treatment success rate after conducting a preliminary validation of IMU-CRP for patients with BPPV.

## Methods

### Study design

**Figure 1:**
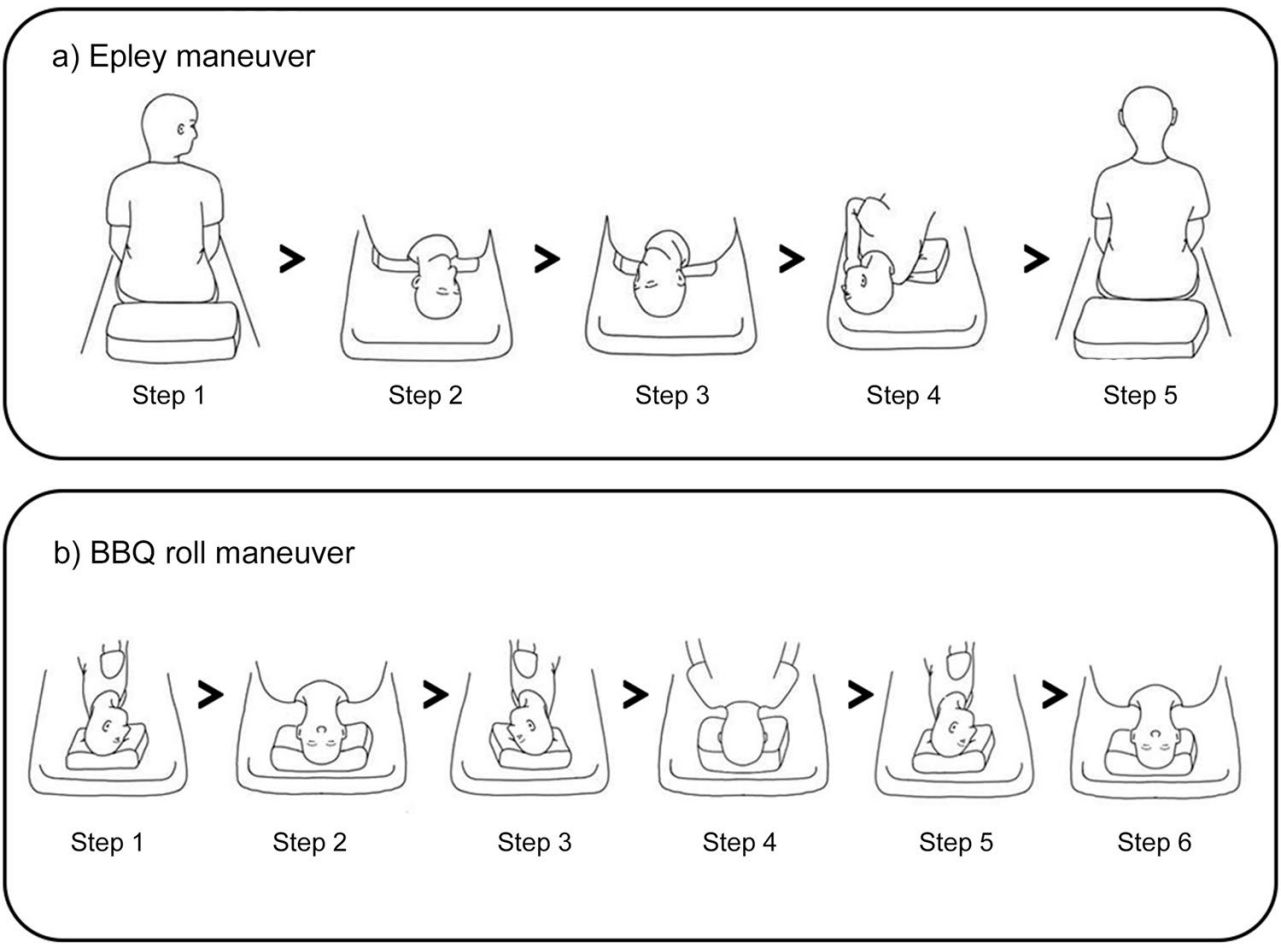
(**a**) Epley maneuver procedure steps. (**b**) Barbeque (BBQ) roll maneuver procedure steps.

Participants performed four sessions each of the Epley and BBQ roll maneuvers^4^. All the participants performed Epley and BBQ roll maneuvers for the right ear. For the Epley maneuver (Fig. 1a), the participants started the maneuver while sitting. In step 1, they turned their head 45° to the right. While keeping this 45° angle, they lay back with shoulders on the pillow, the neck extended, and the head resting on the bed (step 2). Next, they turned their head 90° to the left without raising it (step 3). In step 4, they turned their body 90° to the left while keeping their head angle. In the final step, they sat up (step 5). For the BBQ roll maneuver (Fig. 1b), the subjects started the maneuver while lying in the supine position with the head rotated to the right side and resting on a pillow. In step 1, subjects turned their head 90° to the left (nose pointing to the ceiling) and lowered their head. Next, they turned their head and body 90° to the left again (step 2). In step 3, they turned their head 90° left again, bringing their body into the prone position. In step 4, they turned their head and body 90° to the left. In the last step, their head was turned 90° (supine position) and raised (step 5). For each maneuver, two sessions of the EDU-CRP were followed by two sessions of IMU-CRP. In all eight sessions, subjects wore a laboratory-developed head motion tracking device (HMTD) that quantitatively recorded the head movements in 3-D rotation angles.

For EDU-CRP, subjects were first taught how to perform the Epley maneuver. A single specialist conducted the CRP education in all the participants. A picture handout describing each step was also provided^4^. If questions arose, repeated demonstrations were provided until the participant felt confident about the CRP. Afterward, the HMTD recorded head movements while participants performed the maneuver independently with potential handout guidance. After the first session, the instructor educated the subjects again, explaining incorrect steps and how to improve the CRP. When the subjects had understood this education session, a second trial was performed. The maneuver accuracy was again recorded with the HMTD during the second trial session. No additional education was provided afterward.

For the third and fourth sessions, the IMU provided auditory step-by-step instructions and feedback during the Epley maneuver. As an example of step 1, the initial system instruction was "Please turn your head 45° to the right." If the head rotation angle was too small, the system instructed as follows: “Turn your head a little bit more.” Similarly, if the angle was too large, the system instructed the participant to “Return a little bit toward the original position.” When the head location was within the acceptable range, the system provided audio feedback such as, “This position is good. Stay in this position for 1 min.” The acceptable range of rotation angles was derived from the mean head rotation at each step of the CRP performed by vestibular specialists in a previous study^8^. Auditory feedback was provided with a delay of < 10 ms. The Epley maneuver was performed twice using this system without additional instruction or feedback. After the four Epley maneuver sessions, the BBQ roll maneuver was likewise performed.

In the pilot validation, BPPV patients performed Epley or BBQ roll maneuver guided by our system, and about 1 h after the first maneuver, the absence of both positional vertigo and nystagmus consistent with positional test performed by a neurologist was regarded as successful resolution. Treatment success was defined as the absence of positional symptom and/or negative finding in positional test. When treatment failed, the neurologist performed the same positional maneuver using the IMU device.

### Participants

Between July 2019 and January 2021, we enrolled 19 adults aged ≥ 60 years (mean age ± standard deviation [SD], 62.8 ± 1.9 years; 15 women). Based on a systematic interview or physical examination, it was confirmed that they had no disease or surgery that could induce BPPV and no prior knowledge of BPPV or CRP. In this study we focused on older adults, because this age group has the greatest difficulty in understanding and preforming an accurate CRP^8^. BPPV patients were also enrolled for pilot validation of the treatment efficiency of the IMU-CRP. 10 BPPV patients, who haven’t had head injury or any other disease relevant to dizziness like otitis media, labyrinthitis, and Meniere’s disease, were participated in our study (7 posterior canal BPPV, 3 horizontal canal BPPV, mean age ± standard deviation [SD], 67.1 ± 12.1 years; 8 women).

### Ethics declaration

The Institutional Review Board of Seoul National University Hospital approved this prospective, single-institution study with normal participants (IRB No. H-1412-012-630). Prospective, single-institution study for a pilot validation with BPPV patients was approved by the Institutional Review Board of Myung-ji Hospital (IRB No. MJH-2019-09-021-002). Both studies were conducted in accordance with the ethical standards of the Helsinki Declaration. All participants provided written informed consent.

### Data measurement

**Figure 2:**
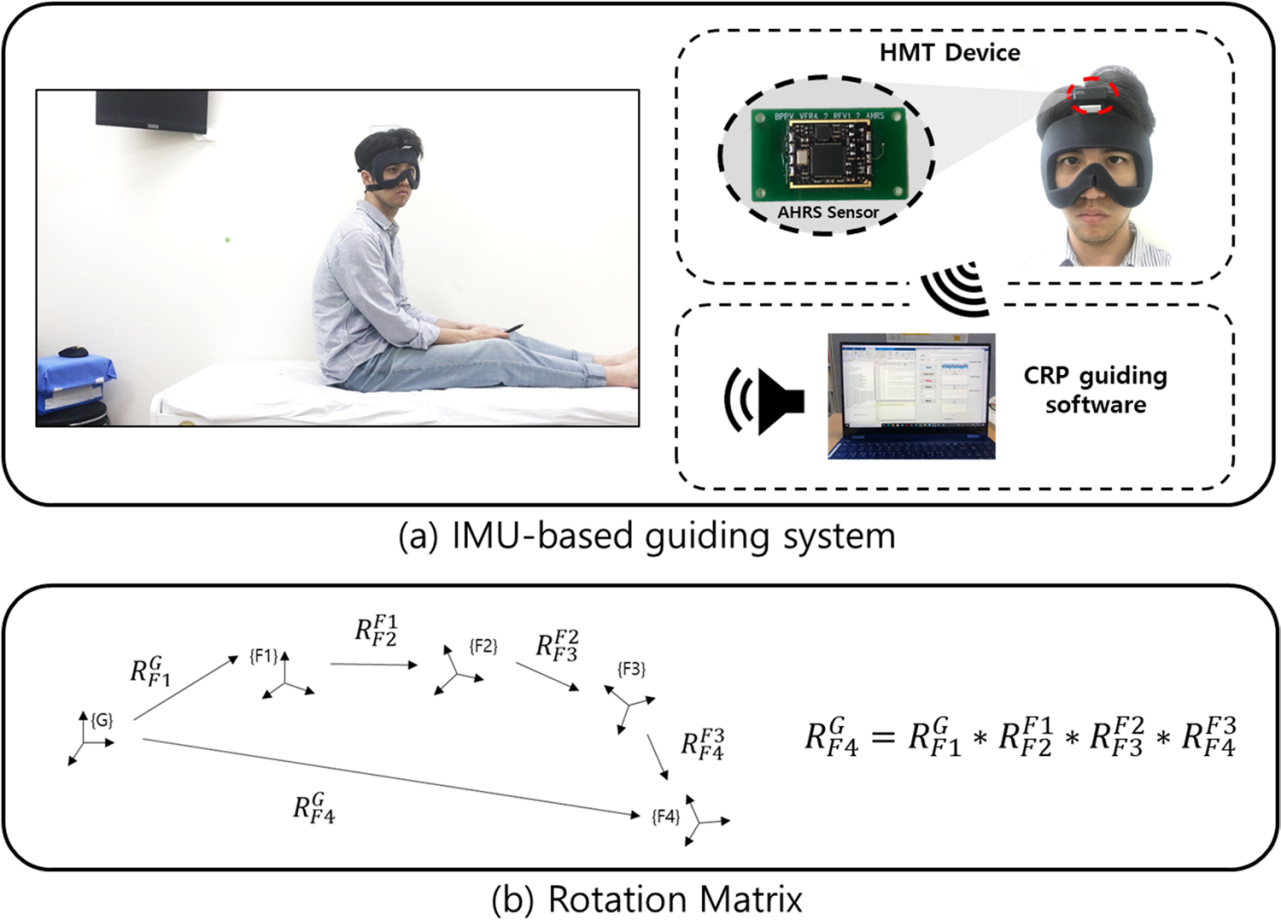
(**a**) The inertial measurement unit (IMU)-based guiding system consists of a head motion tracking (HMT) device and software providing real-time audio guidance for the canalith repositioning procedure (CRP). (**b**) R denotes the rotation matrix.

To measure head motion in real-time, we developed a small (36 × 39 × 14 mm) and lightweight (16 g) HMTD (Fig. 2a). An attitude heading reference system (EBIMU-9DOFV4; E2box, Gyeonggi-do, Korea) was used to track head rotation angles in three axes. Its sensors measured static and dynamic movements in 9 degrees of freedom (3-axis accelerometer, 3-axis gyroscope, 3-axis magnetometer) and determined the 3-axis rotation angles (roll, pitch, yaw), called Euler angles. These angles were transmitted via Bluetooth at a 100-Hz sampling rate to a laptop with the CRP guiding software. The HMTD measured the head motion, and the software calculated the rotation angle to provide auditory feedback.

### Quantitative variables

A rotation matrix was applied to calculate the 3-D rotation angle for each maneuver step from Euler angles^11^. The attitude heading reference system sensor calculated the Euler angles with reference to the earth's center. The rotation matrix calculated the rotation angle relative to an arbitrary axis using the Euler angle. A rotation between two points can be represented by ${R}_{P1}^{P2}$ where p1 and p2 are the start and end points, respectively (Fig. 2b). The multiplication of the rotation matrices for multiple consecutive rotations is identical to a single rotation matrix between the start and end point. We applied this characteristic to calculate the 3-D rotation angle of each step. For example, if the start position is G and the head position at each step is S1, S2, S3, S4, and S5, the rotation matrix for each step (${R}_{G}^{S1}$,${R}_{S1}^{S2}$,${R}_{S2}^{S3}$,${R}_{S3}^{S4}$, and ${R}_{S4}^{S5}$, respectively) can be calculated by multiplying the respective matrices. MATLAB 2017A (MathWorks Inc., Natick, MA, USA) was used to calculate the rotation angles. Calculation of the rotation angle by rotation matrix was validated using a vice, which can fix the object tightly and allow precise rotation. The calculation accuracy was 99.43%.

The details of rotation vector and angle analyses have been reported previously^8^. In brief, we used rotation angle errors (in degrees) to evaluate EDU- and IMU-CRP performances. Errors were defined as differences between actual rotation angles and corresponding target angles specified in the AAO-HNS guidelines^9^. Positive and negative values represent excessive and insufficient head rotation, respectively. The absolute mean error was averaged across all participants.

CRP was considered reliable when the absolute mean error was within the target range defined as the absolute mean error of specialist + half the SD in a previous study^8^, with reference to the AAO-HNS-specified rotation angles. Half the SD was used to represent the minimum difference between groups^12,13^. Thus, target ranges were 26.1°, 31.0°, and 18.9° for steps 2–4, respectively, of the Epley maneuver and 17.2°, 14.6°, 20.1°, and 12.8° for steps 2–5, respectively, of the BBQ roll maneuver.

### Statistical analysis

Due to data loss during the Bluetooth transfer, data of seven participants (Epley maneuver: 3, BBQ roll maneuver: 4) were excluded from further analyses. Data are presented as the mean ± SD. Paired *t* tests of errors and absolute errors between EDU- and IMU-CRPs were performed using SPSS (version 21.0; IBM, Armonk, NY, USA). Paired *t* tests between the first and second sessions per treatment were conducted to evaluate the effects of repetitive trials. Effect size (Glass’s delta) for all the performed paired *t* tests was calculated as well^14^. In a pilot validation with BPPV patients, the treatment success rates of the three outcome measures were calculated for each maneuver.

### Ethics declarations

The Institutional Review Board of Seoul National University Hospital approved this prospective, single-institution study (IRB No. H-1412-012-630), and the first registration took place on 19/06/2019. Prospective, single-institution study for a pilot validation with BPPV patients was approved by the Institutional Review Board of Myung-ji Hospital (IRB No. MJH-2019-09-021-002). Both studies were conducted in accordance with the ethical standards of the Helsinki Declaration. All participants provided written informed consent.

### Consent to participate/consent to publish

All participants provided written informed consent for participation and publication.

## Results

### Accuracies of EDU- and IMU-CRPs

**Figure 3:**
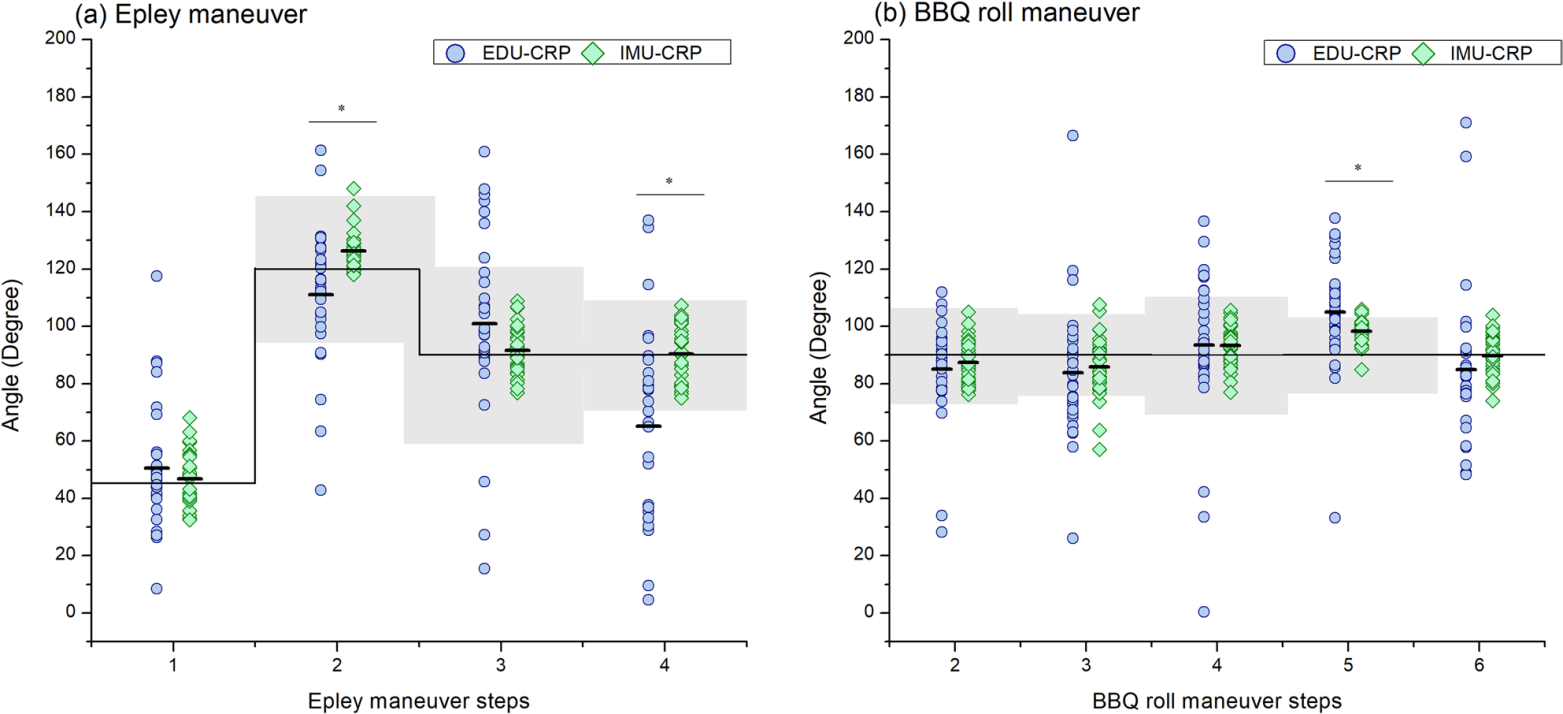
Scatter plots of measured angles in Epley (**a**) and Barbeque (BBQ) roll (**b**) maneuvers. *CRP* canalith repositioning procedure, *EDU* education-based conventional, *IMU* inertial measurement unit.

Figure 3 shows a plot of all measured angles during the Epley and BBQ roll maneuvers. The solid black line represents the target head rotation angle according to the AAO-HNS guidelines. The gray areas indicate target ranges of the head rotation angles derived from specialist-guided CRPs^8^. There are no target range data in step 1 of the Epley maneuver and step 6 of the BBQ roll maneuver due to different start and end points in the specialist CRPs. For the Epley maneuver, the mean errors of steps 1–4 during the EDU-CRP were 5.51 ± 20.43, − 8.98 ± 15.97, 10.89 ± 26.36, and − 24.80 ± 21.84, respectively. During the IMU-CRP, the respective mean errors were 1.87 ± 8.21, 6.25 ± 5.42, 1.62 ± 6.81, and 0.34 ± 9.32. Effect size concerning the mean errors for each Epley maneuver step between EDU-CRP and IMU-CRP were 0.18, 0.95, 0.35, and 1.15, respectively. The mean errors during the IMU-CRP were significantly smaller in steps 2 (*P* = 0.004) and 4 (*P* < 0.001). For the BBQ roll maneuver, the mean errors of steps 2–6 during the EDU-CRP were − 5.03 ± 12.01, − 5.97 ± 18.75, 3.48 ± 21.23, 15.26 ± 14.55, and − 5.05 ± 17.52, respectively. During the IMU-based treatment, the corresponding mean errors were − 2.65 ± 6.46, − 4.07 ± 9.57, 3.25 ± 5.99, 8.25 ± 3.73, and − 0.31 ± 6.48. Effect size concerning the mean errors for each BBQ maneuver step between EDU-CRP and IMU-CRP were 0.20, 0.10, 0.01, 0.48, and 0.27, respectively. The mean error during the IMU-CRP was significantly smaller in step 5 (*P* = 0.049).

**Figure 4:**
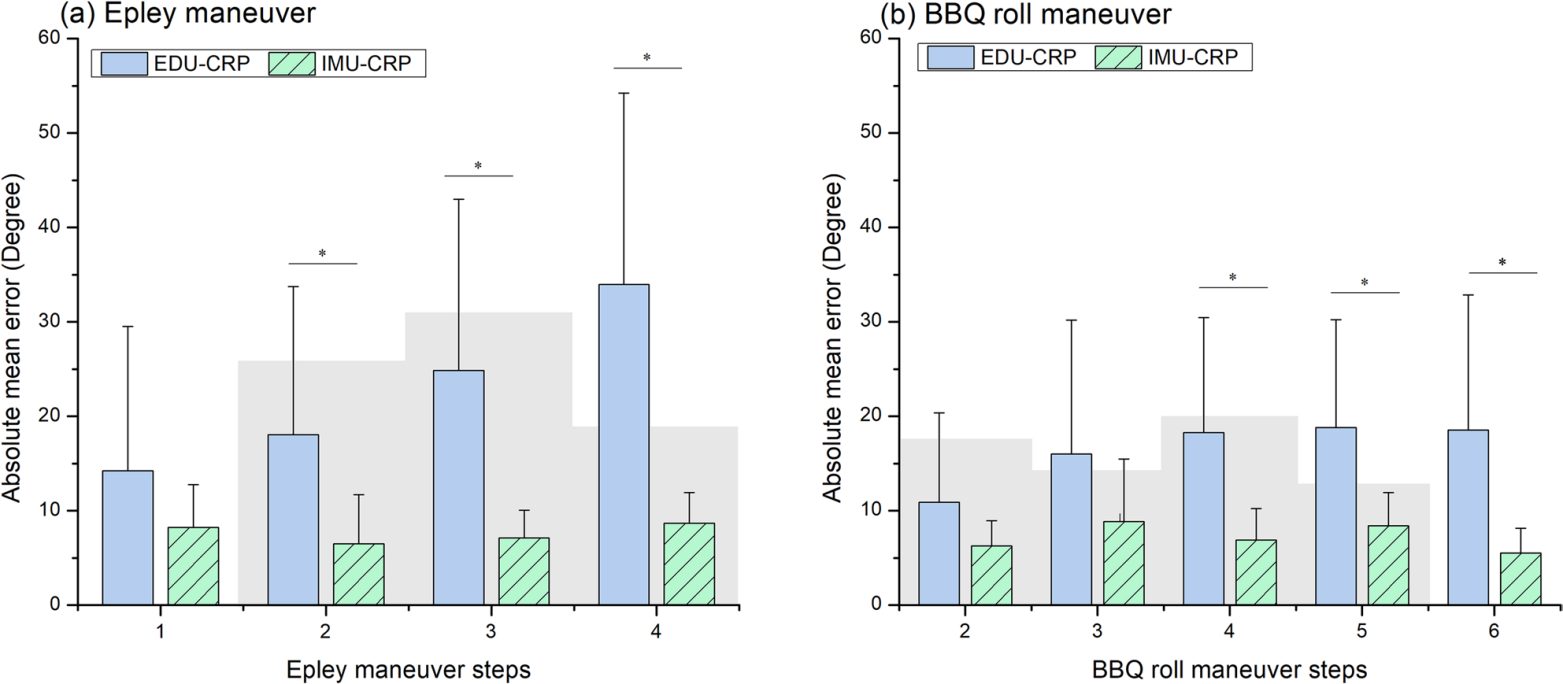
Absolute errors of EDU- and IMU-CRPs in Epley (**a**) and Barbeque (BBQ) roll (**b**) maneuvers. *CRP* canalith repositioning procedure, *EDU* education-based conventional, *IMU* inertial measurement unit.

Figure 4 shows the absolute mean errors of the EDU- and IMU-CRPs for the Epley and BBQ roll maneuvers. The gray areas indicate nominal absolute errors derived from specialist-guided CRPs^8^. For the Epley maneuver, the absolute mean errors of steps 1–4 during EDU-treatment were 14.24 ± 15.28, 18.04 ± 15.71, 24.85 ± 18.12, and 33.98 ± 20.25, respectively. The overall absolute mean error was 22.78 ± 22.14. During the IMU-CRP, the absolute mean errors of steps 1–4 were 8.23 ± 4.52, 6.52 ± 5.18, 7.10 ± 2.96, and 8.67 ± 3.26, respectively. The overall absolute mean error was 7.63 ± 5.43. Effect size concerning the absolute mean errors for each Epley maneuver step between the EDU-CRP and IMU-CRP were 0.39, 0.73, 0.98, and 1.25, respectively. The absolute mean errors during the IMU-CRP were significantly smaller in steps 2 (*P* = 0.012), 3 (*P* = 0.002), and 4 (*P* < 0.001). The percentages of data with absolute mean errors within the target range were 73.3%, 66.7%, and 40.0% during the EDU-CRP and 96.7%, 100.0%, and 100.0% during the IMU-CRP for steps 2–4, respectively.

For the BBQ roll maneuver, the absolute mean errors of steps 2–6 during the EDU-CRP were 10.91 ± 9.43, 16.00 ± 14.21, 18.28 ± 12.19, 18.82 ± 11.41, and 18.55 ± 14.30, respectively. The overall absolute mean error was 16.65 ± 17.13. During the IMU-CRP, the corresponding absolute mean errors were 6.28 ± 2.68, 8.84 ± 6.62, 6.92 ± 3.33, 8.43 ± 3.48, and 5.52 ± 2.62. The overall absolute mean error was 7.22 ± 4.94. Effect size concerning the absolute mean errors for each BBQ maneuver step between the EDU-CRP and IMU-CRP were 0.49, 0.50, 0.93, 0.91, and 0.91, respectively. The absolute mean errors during the IMU-CRP were significantly smaller in steps 4 (*P* = 0.002), 5 (*P* = 0.002), and 6 (*P* = 0.002). The percentages of data with absolute mean errors within the target range were 84.4%, 53.1%, 65.6%, 40.6% during the EDU-CRP and 100%, 84.4%, 100%, and 90.6% during the IMU-CRP for steps 2–5, respectively. All steps considered, the percentages of data with absolute mean errors within the target range were 60.6% and 95.9% for EDU- and IMU-CRPs, respectively.

### Learning effect

**Figure 5:**
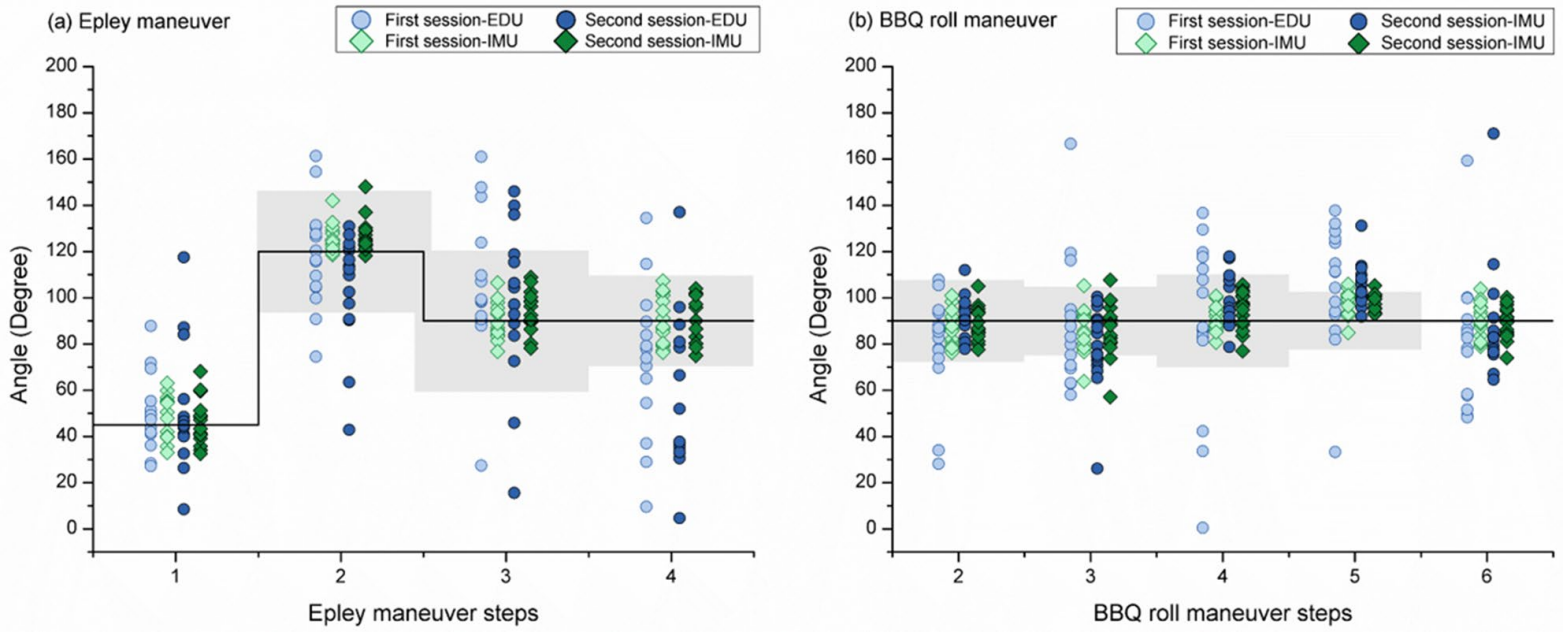
Scatter plots of absolute errors for first and second sessions in EDU- and IMU-CRPs. *BBQ* Barbeque, *CRP* canalith repositioning procedure, *EDU* education-based conventional, *IMU* inertial measurement unit.

Figure 5 shows a comparison between the first and second sessions in EDU- and IMU-CRPs. Solid lines and gray areas correspond to those in Fig. 3. For the Epley maneuver, the absolute mean errors of steps 1–4 during the first EDU-CRP were 11.76 ± 14.16, 17.10 ± 11.85, 23.23 ± 25.57, and 28.30 ± 23.64, respectively. The corresponding data during the second EDU-CRP were 16.73 ± 21.36, 18.98 ± 21.91, 26.47 ± 22.55, and 39.66 ± 25.20, with no significant trial difference, indicating that the learning effect was minimal. Effect size concerning the absolute mean errors for each Epley maneuver step between the first and second session of the EDU-CRP were 0.35, 0.16, 0.13, and 0.48, respectively. The absolute mean error differences between the first two sessions (1st–2nd) were − 4.97 ± 16.10, − 1.88 ± 19.33, − 3.24 ± 31.79, and − 10.60 ± 26.53, respectively. These negative values in every step provide additional evidence of a negligible learning effect.

During the IMU-CRP, the absolute mean errors of steps 1–4 during the first trial were 8.38 ± 4.60, 5.86 ± 5.80, 6.84 ± 4.12, and 9.09 ± 4.77, respectively, whereas those during the second IMU-CRP were 8.08 ± 6.08, 7.18 ± 7.20, 7.37 ± 6.13, and 8.24 ± 4.67, respectively. Again, there was no significant difference between the first and second trials. Effect sizes concerning the absolute mean errors for each Epley maneuver step between the first and second session of the IMU-CRP were 0.07, 0.23, 0.13, and 0.18, respectively. The absolute mean error differences between the two sessions were 0.31 ± 5.87, − 1.32 ± 7.98, − 0.53 ± 8.61, and 0.85 ± 6.82. All four values approached zero.

For the BBQ roll maneuver, the absolute mean errors of steps 2–6 during the first EDU-CRP were 15.11 ± 18.23, 18.73 ± 19.13, 26.40 ± 24.33, 24.26 ± 16.85, and 20.65 ± 19.40, respectively. The corresponding mean errors during the second EDU-CRP were 7.00 ± 5.68, 13.65 ± 15.34, 10.85 ± 9.45, 14.04 ± 10.18, and 15.77 ± 18.91, respectively. The absolute mean error of the second EDU-CRP was significantly smaller in steps 4 (*P* = 0.039) and 5 (*P* = 0.026). Effect sizes concerning the absolute mean errors for each BBQ maneuver step between the first and second session of EDU-CRP were 0.44, 0.27, 0.64, 0.61, and 0.25, respectively. The absolute mean error differences between the first two sessions (1st–2nd) were 8.11 ± 19.45, 5.08 ± 19.90, 15.56 ± 27.52, 10.23 ± 16.37, and 4.87 ± 25.88; thus, repeated education improved the performance of the BBQ roll maneuver by 15.56° in step 4 and 10.23° in step 5.

During the IMU-CRP, the absolute mean errors of steps 2–6 were during the first trial, 6.62 ± 4.33, 7.83 ± 6.73, 5.07 ± 3.46, 8.73 ± 4.01, and 5.82 ± 4.10, respectively, and during the second trial 6.44 ± 3.76, 9.31 ± 8.00, 8.25 ± 4.74, 8.40 ± 3.61, and 5.71 ± 3.98, respectively. The absolute mean error of the second IMU-CRP was significantly larger in step 4 (*P* = 0.022). Effect sizes concerning the absolute mean errors for each BBQ maneuver step between the first and second session of the IMU-CRP were 0.04, 0.22, 0.92, 0.08, and 0.03, respectively. The absolute mean error differences between the two sessions were 0.19 ± 5.79, − 1.48 ± 6.29, − 3.18 ± 4.97, 0.33 ± 3.51, and 0.11 ± 5.25; thus, the performance of the BBQ roll maneuver worsened after repetition of IMU-CRP by 3.18° in step 4.

### Treatment efficiency

**Figure 6:**
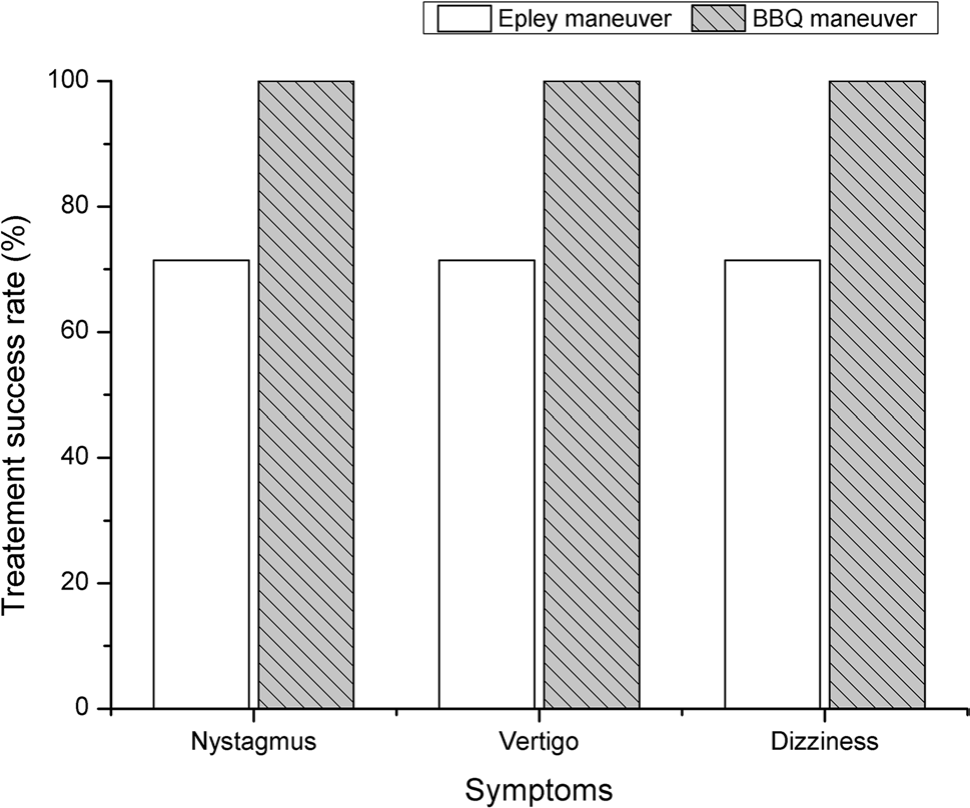
Treatment success rates of the IMU-CRP in BPPV patients. *BBQ* Barbeque, *CRP* canalith repositioning procedure, *IMU* inertial measurement unit.

Figure 6 shows the treatment success rates of the IMU-CRP in BPPV patients. The treatment success rate of the Epley maneuver was 71.4% (5 out of 7 patients), while that of the BBQ maneuver was 100% (3 out of 3 patients).

## Discussion

The present study aimed to validate the feasibility of self-administered CRPs assisted by an IMU sensor-based guiding system. This study found a significant benefit of this guiding system. The (absolute) mean errors in IMU-CRPs were significantly smaller than those of conventional EDU-CRPs in both Epley and BBQ roll maneuvers. Moreover, the IMU-based system can assist patients in performing CRP at home as accurately as professionals in the clinic. In 95.9% of patients, the absolute mean errors of IMU-CRPs were within the target range derived from vestibular specialists with > 10 years of clinical experience. Repeated CRPs improve treatment outcomes in BPPV and prevent symptom recurrence^4–7^. However, EDU-CRP does not seem reliable: in the Epley maneuver, (absolute) mean errors were substantial despite repeated education, and the angular head rotation was within the target range of only 60.6%. Seemingly, participants can learn the concept of the Epley maneuver, but precise head rotation is difficult to achieve through education. Real-time feedback from the proposed IMU-based system seems key to enabling participants to perform Epley and BBQ roll maneuvers on their own accurately.

The feedback system seems to be the most important difference between EDU- and IMU-CRPs. Education and feedback are both instructions on how to perform CRP correctly. Education describes the general approach, whereas feedback enables correction of the current head position. Even if participants perfectly understood each step, they might have difficulties executing the learned CRP. Since the IMU system instructs the participant to move the head until it is within the target range, the participant will eventually achieve the correct position without fully understanding the entire procedure. Therefore, IMU sensor-based guiding system can be an effective adjunct to CRP treatment.

Several studies described in-home systems for BPPV treatment. A device called DizzyFix™ has been approved by the US Food and Drug Administration^15^. This cap-attached device guides the patient to navigate a ball within the device through head movements. These authors also developed a mobile app for an Epley maneuver guiding system^16^. A virtual reality-based system has also been proposed^17^. These systems were only evaluated by physicians subjectively rating CRP performances. Moreover, these systems do not provide real-time quantitative feedback on head motions. Recently, another research group developed an Epley maneuver visual assistive device^18^. In this study, the author performed a quantitative analysis of rotation error when performing the Epley maneuver with and without the assistive device. The result was consistent with our research finding. However, this visual assistive device helps the clinician to treat the patient more easily and does not help the patient perform the Epley maneuver by themselves more accurately. Our IMU-based wearable system computes the rotation angle in real-time and provides audio guidance to achieve AAO-HNS targets. It can be applied to Epley and BBQ roll maneuvers, the latter of which has not been addressed previously. Horizontal canal BPPV may be more common than posterior canal BPPV, depending on the evaluation time^19–22^. Moreover, the adaptability of our guiding system allows the addition of any treatable BPPV subtype.

As seen in Fig. 5, there was no significant difference between the first and second session of absolute error angle in the EDU-CRP of Epley maneuver, which indicates that the traditional education-based feedback was not effective. Meanwhile there was a significant difference between the EDU-CRP and IMU-CRP. We did not provide any additional education, feedback of previous EDU-CRP, or practice between the EDU-CRP and IMU-CRP. If the repetition of CRP has a practice effect, we expect the absolute error angle to decrease gradually after each session. However, the absolute mean error did not change after repeated EDU-CRP but dramatically decreased only when IMU-CRP was performed (Fig. 4). This result implies that real-time CRP feedback (IMU-CRP) and not traditional education or repeated practice is responsible for the difference. We hypothesize that it is difficult to validate head rotation internally without external real-time guidance with the head reclined. Moreover, changing the body position during step 4 may distract from precisely controlling the head rotation. External feedback can help overcome these problems. The suggested guiding system seems to facilitate this. Since education does not provide real-time feedback, the benefit may be limited in complex CRPs such as the Epley maneuver. By contrast, the BBQ roll maneuver is geometrically simple, and participants can easily obtain environmental cues. Education alone may be sufficient for simple CRPs such as the BBQ roll maneuver. According to this study, the absolute mean error significantly decreased during the second EDU-CRP in steps 4 and 5 of the BBQ roll maneuver. Although the Epley maneuver is more complex than the BBQ roll maneuver, our IMU-based system is effective in both maneuvers.

According to Song et al., the resolution rate of positional nystagmus and vertigo in patients with posterior canal BPPV was 67.6% at post-1 h^23^. Our IMU-based guiding system has potential as a BPPV treatment device, as it showed a treatment success rate of 80% after 1 h in a pilot validation for 10 BPPV patients.

The present study has several limitations. First, effects of inaccurate head rotations on treatment outcomes were evaluated only with a small number of BPPV patients for preliminary validation. Further studies are required to validate the treatment outcomes of this IMU-based system for vertigo and nystagmus in more patients with BPPV. It may be difficult for some patients with BPPV to properly carry out the instructions when nystagmus and symptoms occur. Thus, we recommend using this system after the diagnosis and initial treatment by a medical personnel at the hospital (under the classic medical care system) to avoid misdiagnosis, provide initial CRP education to the patient, and reduce symptoms to enable the patient to perform CRP alone. Second, age and sex effects on guiding effectiveness were not considered because of the limited number of study participants. We recruited only participants aged ≥ 60 years for three reasons: (1) BPPV is more prevalent in elderly participants^9,24,25^. (2) Younger participants have less difficulty learning geometrically complex 3-D head movements^8^. (3) Recurrence and residual symptoms requiring additional management are more frequent in elderly participants^26–28^. Although clinically irrelevant, the study results may change if young participants were exclusively recruited.

## Conclusion

Real-time feedback on head rotation angles by an IMU sensor-based guiding system enabled the participants to perform Epley and BBQ roll maneuvers on their own accurately. IMU-CRP accuracy was comparable to that of vestibular specialists. Repeated self-administered CRPs have been reported to improve treatment outcomes and prevent BPPV recurrence; however, according to the present study results, education-based conventional self-administered CRP is unreliable. The IMU-based system can be useful in elderly patients with BPPV with recurring or residual symptoms by helping them repeat accurate CRPs independently, even when a vestibular specialist is unavailable.

## Data Availability

The datasets generated during and/or analyzed during the current study are not publicly available due to privacy concerns and violation of agreement of the informed consent process, but the datasets are available from the corresponding author on reasonable request.
